# The relationship between the force applied and perceived by the surgeon during ureteral access sheath placement: ex-vivo experimental model

**DOI:** 10.1007/s00345-024-04982-7

**Published:** 2024-05-16

**Authors:** Tzevat Tefik, Rifat Burak Ergül, Palle Osther, Guido Giusti, Glenn M. Preminger, Michael Straub, Jens Jochen Rassweiler, Emanuele Montanari, Marianne Brehmer, Christian Seitz, Michael Grasso, Margaret Pearle, Silvia Proietti, Jonathan Cloutier, MGuven Gunver, Ismet Nane, Faruk Ozcan, Olivier Traxer

**Affiliations:** 1 Department of Urology, Istanbul University Istanbul Faculty of Medicine, Istanbul, Turkey; 2 Progress in Endourology, Technology and Research Association (PETRA), Paris, France; 3 Department of Urology, Urological Research Center, Lillebælt Hospital, University Hospital of Southern Denmark, Vejle, Denmark; 4 Department of Regional Health Research, University of Southern Denmark, Odense, Denmark; 5 Department of Urology, IRCCS San Raffaele Hospital, Milan, Italy; 6 Department of Urology, Duke University Medical Center, Durham, USA; 7 Department of Urology, Technical University Munich, Munich, Germany; 8 Department for Urology and Andrology, Danube Private University, Krems, Austria; 9 Department of Urology, Fondazione IRCCS Ca’ Granda Ospedale Maggiore Policlinico, Milan, Italy; 10 Department of Clinical Sciences and Community Health, University of Milan, Milan, Italy; 11 Department of Urology, Stockholm South General Hospital Stockholm, Karolinska Institutet, Stockholm, Sweden; 12 Department of Clinical Science, Stockholm South General Hospital Stockholm, Karolinska Institutet, Stockholm, Sweden; 13 Department of Urology, Comprehensive Cancer Center, Medical University of Vienna, Vienna, Austria; 14 Department of Urology, Phelps Hospital/Northwell Health, New York, USA; 15 UT Southwestern Medical Center, Dallas, TX USA; 16 Division of Urology, Department of Surgery, CHU de Québec-Université Laval, Quebec, QC Canada; 17 Department of Medical Statistics, Istanbul Faculty of Medicine, Istanbul University, Istanbul, Turkey; 18 GRC N°20, Groupe de Recherche Clinique sur la Lithiase Urinaire, Hôpital Tenon, Sorbonne Université, 75020 Paris, France

**Keywords:** Retrograde intrarenal surgery, Tactile sensation, Ureteral access sheath, Peak force of insertion, Ureteral injury

## Abstract

**Purpose:**

To define a peak force of insertion (PFOI) threshold for ureteral damage during ureteral access sheath (UAS) placement on an experimental ureteral orifice model.

**Methods:**

A specially designed water tank using 2 laparoscopic 5 mm ports and 2 different size (10 Fr and 8 Fr) sealing cap adaptors (SCA) as ureteral orifices was used to perform the test. A 10–12 Fr UAS was fixed to a load cell and the force of insertion (FOI) was continuously recorded with a digital force gauge.13 experts in the field of endourology who participated performed 3 UAS insertions. The FOI was recorded initially with 10 Fr followed by 8 Fr SCA. On the final insertion, the orifice was obstructed, leaving a 5 cm length to insert the UAS. The experts were asked to “Stop at the point they anticipate ureteral damage, and they would not proceed in real life”.

**Results:**

Using 10 Fr SCA the PFOI was 2.12 ± 0.58 Newton (N) (range:1.48–3.48) while 8 Fr SCA showed a PFOI 5.76 ± 0.96 N (range:4.05–7.35). Six of the experts, said they would stop proceeding when they reached above 5.1 N. Three experts had PFOI < 5.1 N and the other 4 stated they would go with PFOIs of 5.88, 6.16, 6.69 and 7.35 N when using SCA of 8 Fr.The highest load they would stop proceeding had a PFOI of 6.09 ± 1.87 N (range: 2.53–10.74).

**Conclusion:**

The PFOI threshold for ureteral damage inserting UAS of the experts is variable. Although FOI is a subjective perception, experience suggests that ureteral injury may occur at an average of 6.05 N perceived by surgeons’ tactile feedback. In-vivo measurement of UAS PFOI may confirm a threshold.

## Introduction

In recent years, retrograde intrarenal surgery (RIRS) has become an important option in the treatment of upper urinary tract stones [1]. The standard use of UAS when treating a stone in RIRS has been a matter of debate and is used by most urologists in appropriate cases [2]. UAS popularity has grown due to the numerous potential advantages it offers. These include facilitating access to the renal collecting systems, enabling multiple entry and reentry points, lowering intra-renal pressure, passively eliminating small stone fragments, and improving drainage around the scope [3–6]. However, it is important to note that the use of the UAS itself carries certain risks. Over-distension of the ureter during UAS insertion can lead to ureteral damage, compromising blood flow and potentially causing ureteral ischemia [7]. The insertion process itself may also pose a direct risk to the ureter, potentially resulting in injury [8].

Insertion of a UAS into the ureter depends on the coefficient of friction of the sheath surface and axial forces that may cause the sheath to buckle at the ureteral orifice [9]. There are a few studies that measure the external PFOI applied by the surgeon for UAS insertion, and the fusion of these instantaneous force-measuring devices into the UAS are being developed. However, in daily practice in a challenging ureter, the surgeon decides the maximum force to apply with tactile sensation. This PFOI may guide surgeons on whether to proceed with UAS when the risk of ureteral injury is anticipated.

The aim of this study was to measure the PFOI that would stop the procedure during UAS placement by expert endourologists on a homemade model with a digital force gauge.

## Materials and methods

**Fig. 1:**
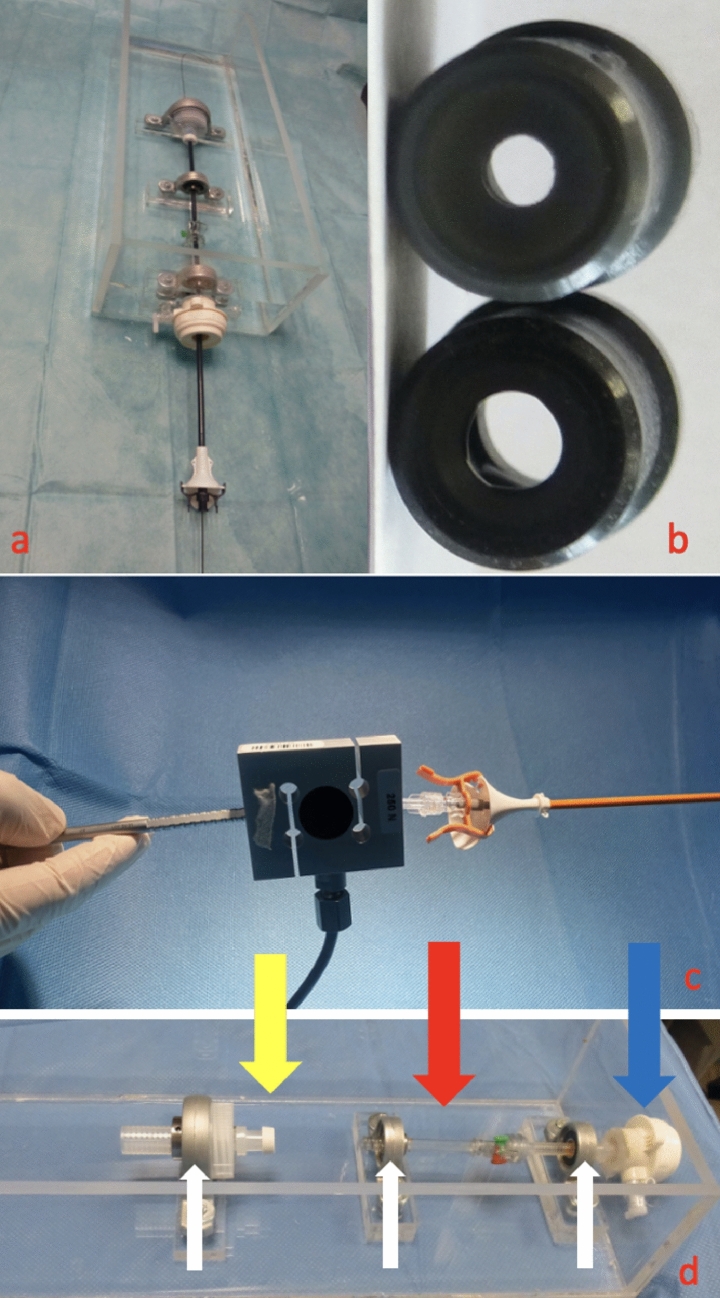
**a** Ex-vivo experimental home water-tank model (View of the tank from top) **b** a 10 and an 8 Fr sealing cap adaptor used as an ureteral orifice, **c** force gauge adapted to an ureteral access sheath, ready for insertion. **d** Lateral view of the water tank, Blue Arrow: First Laparoscopic port, Red Arrow: Second Laparoscopic port, Yellow Arrow: Adapter, White Arrows: Metallic Stabilizers

A special water tank (Fig. 1a and d) was designed to insert the UASs into an adapter (ex-vivo ureteral orifice) with 2 different diameters of 10Fr and 8Fr (Fig. 1b). Two 5 mm laparoscopic ports were longitudinally connected with each other and fixed in the water tank with metallic stabilizers to prevent UAS buckling. (Fig. 1d) A 10–12 Fr UAS was fixed to a load cell and the insertion force was continuously recorded with a digital force gauge (DFS II, Chatillon®, Ametek® Test and Calibration Instruments, Largo, Florida, USA) during wet insertion in the water tank in an approximate constant speed of 25 mm/s. (Fig. 1c) The PFOI measurements were done after evaluating the preliminary results of the experiment, where similar force measurements were achieved using the same UAS. Each of the 13 experts in the field of endourology, with large RIRS experience and UAS placement performed 3 UAS insertions. The FOI was recorded with 10 Fr followed by an 8 Fr adapter imitating the ureteral orifice. On the final third insertion, the orifice was obstructed, leaving a 5 cm length to insert the UAS. Participants were instructed to proceed until they felt a level of resistance that would stop them if they were inserting an UAS in a real patient. They were blind to the real-time force measurements. Participants were asked to perform the experiment under the same conditions on a 2-day conference meeting.

### Statistical analysis

Statistical analysis was performed in SPSS version 28. One-way ANOVA was applied to determine the difference between peak forces among accesses. In addition, Tukey’s HSD post hoc test was applied to determine superiority between accesses. Statistical significance level (alpha) was set to 0.05.

## Results

For 13 expert urologists, using 10 Fr SCA the peak and average FOI was 2.12 ± 0.58 N (range: 1.48–3.48) and 0.76 ± 0.44 N (range: 0.04–1.54) while 8 Fr SCA showed a peak and average FOI of 5.76 ± 0.96 N (range: 4.05–7.35) and 2.77 ± 1.37 N (range: 0.37–5.79), respectively. (Table 1) Six of the experts said they would stop proceeding when they reached above 5.1 N. Three experts had PFOI < 5.1 N and the other 4 said they would go with PFOIs of 5.88, 6.16, 6.69 and 7.35 N when using SCA of 8 Fr.

In the orifice occluded model, the highest load they would stop proceeding had an average PFOI 6.09 ± 1.87 N (range: 2.53–10.74). When excluding the 2 experts on the extreme of the PFOIs, the highest load to stop proceeding had a PFOI of 6 ± 0.87N (range: 5.41–7.65). Five of the participants (M.S., J.R., MG, C.S., and J.C) stopped at a PFOI lesser than that of 8 Fr SCA in Test 3.

Test 1 had statistically significantly lower PFOI values compared to test 2 and 3 (*p* < 0.001). None of the participants recorded PFOI > 3.5 N and none reported to stop inserting the UAS in test 1. While the average PFOI was 5.76 N and 6.09 N in test 2 and 3, respectively, without any statistical significance.

## Discussion

The adapter diameter in test 1 was 10 Fr and is actually wider than the normal ureteral orifice [10]. The adapter diameter in test 2 was 8 Fr, with an average PFOI of 5.76 N. In test 3, the average PFOI that expert surgeons would stop in a real operation is 6.09 N, and in fact, the average PFOIs are close to each other in tests 2 and 3. This is a finding that may support the use of an 8 Fr adapter diameter as an orifice model in future experiments.

It is generally assumed that the cause of ureteral injuries caused by UAS is secondary to excessive insertion force. In the study by Tapiero et al., they showed that the PULS score increased with the increase in PFOI, independent of the UAS diameter [11]. They also showed that a 1 N increase in PFOI was associated with a 0.07 increase in PULS grade (*p* = 0.01). In the study, it was suggested that if the PFOI exceeds 6 N, no more force should be applied and that re-placement of the UAS should be attempted by reducing the UAS diameter. However, in the study, PFOI exceeded 6 N in 121 (57%) procedures and 8 N in 39 (19%) procedures. The highest PFOI recorded was 12.4 N. Pulse 3 injuries occurred in 2 patients. One of them had a PFOI of 8 N and the other of 8.4 N. In our study, we identified the PFOIs that expert endourologists would discontinue UAS placement. The PFOI, which expert endourologists would stop with tactile sense, was 10.7 N at the highest and 2.5 N at the lowest, representing a wide range. When we dismiss the highest and lowest PFOIs, the mean PFOI was 6 ± 0.87 N. In fact, this supports the suggestion of Tapiero et al. to reduce the UAS diameter if the PFOI exceeds 6N. Except for one expert endourologist, the PFOI did not exceed 8 N.

In their experimental study, Fukui et al. showed that the tip-to-base flexibility ratio and frictional force of PFOIs were mainly related to the insertion force [12]. In light of these findings, the PFOIs applied when placing UAS of different brands in the same ureter may be different. However, in our study, we determined the PFOIs that expert surgeons would discontinue during an operation in a closed system. We think that these determined PFOIs will not vary in the majority of cases using different diameters and UAS brands. However, the degree of flexibility of UASs may affect the PFOIs that surgeons will not proceed with placement. Therefore, we anticipate that further studies are needed in this area.

In a previous study, we measured FOIs continuously in 7 female patients during UAS placement with the same force gauge used in this experiment [13]. In one patient, we found a PFOI of 5.9 N. At the end of the procedure, a superficial bleeding tear in the intramural ureteral orifice was seen under direct vision. In fact, the expert surgeons in this study met upon a mean PFOI of 5.76 N, which they stopped with their tactile feedback. Considering the experience of expert surgeons, we may state ‘’experts’ hands seem to be as precise as a force gauge". However, instantaneous visualisation of these FOIs may prevent a possible trauma that may occur due to the overconfidence of any surgeon.

Pedro et al. simulated in an experimental setting the maximum force to be applied by urologists (with an average of 4.9–9.3 years of experience with UAS and no fellowship in endourology) and residents (second to fourth year in urology residency training) [14]. Urologists had a mean PFOI of 6.55 ± 0.45 N, while residents had a mean PFOI of 4.84 ± 0.64 N. There was a significant difference between the groups (*p* = 0.035). Probably the lack of experience of the residents led them to be more cautious. In fact, the use of this model in urology residency training may provide residents with more confidence when placing a UAS.

Koo et al. measured the force applied during UAS insertion in the human ureter for the first time [15]. They did not report any ureteral injury (grade 2 and above) in cases with PFOI below 5.88 N during UAS placement. In fact, this is very close to the average PFOI of 6.09 N in this study among expert urologists. They also showed that alpha-blockers used in the preoperative period reduced PFOI. Moreover, there are many parameters affecting PFOI such as patient-related alpha-blocker use, history of previous ureteral operation, JJ stenting, history of ureteral stricture, gender and muscle mass. Our study was an experimental study and ignored patient-and tissue-related factors. Ureteral injury during retrograde access has actually many unidentified parameters. It probably has multifactorial causes, and these do not only depend on PFOI during UAS placement. Some ureters are more compliant and resistant bearing higher PFOIs whereas others are more fragile, getting damaged with low PFOIs [15].

Kaler et al. investigated the effect of force applied on pigs evaluating PULS scores [16]. When PFOI remained < 4.84 N, they did not observe any significant injury, whereas, for PFOI exceeding 8.1 N the PULS ≥ 3 injuries were observed routinely. They also showed that safe passage up to 5.56 N is allowed with serial dilation. Our study, was performed in an ex-vivo environment had average PFOI of 6.09 N for safe insertion perceived by the expert urologists but the effect of serial dilation was ignored. Jiang et al. investigated the effect of preoperative stent placement on the UAS diameter in a pig model [17]. Based on the work of Kaler et al., they exerted force < 6 N with serial ureteral dilators and reported a 3.8 Fr increase in ureteral luminal circumference with PULS grade $\le$ 2 with only one ureter having PULS grade 2. This supports our perceived safe PFOI 6.09 N for a pig model with PULS grade $\le$ 2, too.

Graversen et al. investigated the effect of safety wire (SW) use on PFOIs occurring during UAS insertion in a porcine model [18]. They reported that the use of SW significantly increased PFOI but had no effect on the degree of ureteral injury. Monga et al. investigated buckling and bending resistance and lubricity in different UASs. In their study, The Cook Flexor sheath was more resistant to buckling, and both the Cook Flexor sheath and Applied access sheath were more lubricious than the other sheaths tested [9]. Many factors such as SW use, UAS brand and diameter, hydrophilicity, and friction coefficient, which depend on the choice of the urologist, affect PFOI. In our study, a single UAS was used, and other variables were excluded.

This study has certain limitations. Since this was an in vitro study, it could not imitate an exact human ureteral orifice nor ureteral contraction of the smooth muscle wall. Furthermore, could not replicate the resistance along the urethra, urethral sphincters, and ureteral narrowings. Hence, the impact of the urethral resistance and segment of the ureter above the orifice was not part of the model. Another limitation is that the model is not designed to replicate an exact clinical situation, but it is merely to compare expert force perception with different orifice models. A graph of the UAS insertion force was plotted using an experimental model and PFOI were recorded. In this experimental model, surgeons were told to proceed until they felt a level of resistance that would theoretically harm the ureter and they would stop proceeding though surgeons may have applied different forces than in a real-life scenario. Five participants exerted less PFOI for the occluded model compared to the 8Fr SCA, which suggests that the initial two insertions might have sensitized the experts and thus became more aware of placing the 10–12Fr UAS when passage would not have been possible when the point of entry was occluded. The main objective of this study was to determine the PFOI to discontinue UAS placement and was presented in this experimental setting. However, with the stress of surgery on the human ureteral orifice, surgeons may stop earlier or force more. Therefore, with the upcoming devices in the future, it might be reasonable to use force recordings to safely place UASs.

We think that urology resident endourology training programmes should include stations such as PFOI measurements during UAS placement. Our study is an example of such an experimental model, and we believe that will provide both a short learning curve and less complication rates with the objective data it would provide.

**Table 1:** The peak and average FOI of the expert urologists

		P.O	G.G	G.P.	M.S	J.R	E.M.	M.B	C.S	M.G	M.P.	J.C.	S.P.	O.T	AVERAGE
Test 1 (10 Fr)															
PFOI		1.48	1.52	1.78	1.66	2.16	1.5	1.91	3.48	2.06	2.4	2.44	2.69	2.42	2.12
AFOI		0.34	0.58	0.59	0.88	0.31	0.74	0.85	1.54	0.04	0.49	0.94	1.42	1.18	0.76
Test 2 (8 Fr)															
PFOI		4.05	5.88	4.83	6.04*	5.81*	6.76*	4.48	6.16	6.39*	5.12*	6.69	5.28*	7.35	5.76
AFOI		1.47	2.32	1.61	3.05	2.75	3.29	1.5	3.78	0.37	5.79	3.68	3.09	3.36	2.77
Test 3															
PFOI		5.41	7.65	5.88	2.53	5.04	7.39	5.12	5.48	5.56	5.79	6.14	6.5	10.74	6.09
AFOI		3.77	4.33	3.06	1.54	2.57	4.97	2.48	2.3	0.42	1.81	4.10	3.51	6.64	3.19

*PFOI* peak force of insertion, *AFOI* average force of insertion

*Participants stated that they would not proceed at the recorded PFOI

## Conclusions

The 13 expert urologists recorded a PFOI with an average of 6.09 ± 1.87 N to stop inserting a UAS to prevent ureteral injury. It is noteworthy that previously published human in vivo studies reported ureteral injury with a PFOI greater than 6 N, which was similar to the PFOI of the current study. This indicates that experience is important for UAS insertion to prevent ureteral injury. An educational model showing PFOI might guide junior endourologists to perceive the force of their hands during UAS insertion. Finally, measurement of UAS insertion force and reporting ureteral injury in real-life cases will confirm the PFOI threshold.

## Data Availability

Data available on request from the authors. The data that support the findings of this study are available from the corresponding author, upon reasonable request.
